# Keeping up with evidence-based recommendations – a qualitative interview study with general practitioners in Germany on information-seeking behaviour in cardiovascular care

**DOI:** 10.1186/s12875-023-02069-7

**Published:** 2023-05-25

**Authors:** Christine Arnold, Patrick Hennrich, Michel Wensing, Charlotte Ullrich

**Affiliations:** 1 Department of General Practice and Health Services Research, Heidelberg University Hospital, Im Neuenheimer Feld 130.3, 69120 Heidelberg, Germany; 2 Division of Neonatology, Department of Paediatrics, Inselspital, Bern University Hospital, University of Bern, Friedbühlstrasse 19, Bern, 3010 Switzerland; 3 Section for Translational Health Economics, Department for Conservative Dentistry, Heidelberg University Hospital, Heidelberg, Germany

**Keywords:** Ambulatory cardiology care, Cardiovascular diseases, Coordination of care, Germany, Information-seeking behaviour, Information behaviour, Information practice, Primary care, Qualitative interviews

## Abstract

**Background:**

Due to the nature of their work, general practitioners (GPs) need to be up to date with evidence in various medical domains. While much synthesised research evidence is easily accessible nowadays, in practice, the time to search for and review this evidence proposes a challenge. In German primary care, the knowledge infrastructure is rather fragmented, leaving GPs with relatively few primary care specific resources of information and many resources from other medical fields. This study aimed to explore GPs information-seeking behaviour regarding evidence-based recommendations in cardiovascular care in Germany.

**Methods:**

To explore views of GPs a qualitative research design was chosen. Data was collected through semi-structured interviews. In total, 27 telephone interviews with GPs were conducted between June and November 2021.Verbatim transcript interviews were then analysed using thematic analysis, deriving at themes inductively.

**Results:**

Two broad strategies of information-seeking behaviour in GP could be distinguished: (a) generic information-seeking behaviour and (b) casuistic information-seeking. The first referring to strategies GPs apply to keep up with medical developments such as new medication and the second referring to purposeful information exchange regarding individual patients, such as referral letters. The second strategy was also used to keep up with medical developments in general.

**Conclusion:**

In a fragmented information landscape, GPs used information exchange on individual patients to remain informed about medical developments in general. Initiatives to implement recommended practices need to take this into account, either by using these sources of influence or by making GPs aware of possible bias and risks. The findings also emphasize the importance of systematic evidence-based sources of information for GPs.

**Trail registration:**

We registered the study prospectively on 07/11/2019 at the German Clinical Trials Register (DRKS, www.drks.de) under ID no. DRKS00019219.

**Supplementary Information:**

The online version contains supplementary material available at 10.1186/s12875-023-02069-7.

## Background

Due to the nature of their work, general practitioners (GPs) need to be up to date with evidence in various medical domains. Especially when confronted with new developments in treatment, diagnosis and medication, they have to develop strategies to obtain the information needed [1]. While much synthesised research evidence – as presented by systematic reviews and clinical guidelines – is nowadays easily accessible on the internet, in practice, actually finding the time to search for, select and read information is a major challenge. A lack of relevance in daily primary care practice of the available guidance for decision is another obstacle [2]. Therefore, not so much accessibility but selection and prioritisation of guidance for general practice are central in the uptake of new evidence. At the same time, most clinicians employ habitualised illness scripts, also described as “mindlines” [3], when diagnosing and treating a health problem [4]. A study on virtual communities of physicians showed, that this kind of knowledge can be characterised as “knowledge-in-context” with a strong focus on casuistry [5].While in some countries, such as the United Kingdom, the Netherlands or Denmark, vocational training and continuing education in primary care are well-developed and physicians mainly base decisions on primary care guidelines and other evidence-based sources, in other countries, such as Germany, profession-specific systematic information sources are scarce and in development [6]. Therefore, in German primary care, the knowledge infrastructure is rather fragmented, leaving GPs with relatively few primary care specific resources of information and many resources from other fields. A study on participation in coordinated ambulatory cardiology care, for example, showed, that GPs in Germany tend to refer to guidelines from other medical disciplines – however, the perceived relevance for primary care is heterogenous and may be limited [7, 8].

Regarding the uptake of information, two aspects can be distinguished: (a) information-transfer or dissemination strategies by senders and (b) information-seeking behaviour of recipients. *Dissemination strategies* are usually divided into mass media (e.g. written material such as scientific journals, newsletter or internet-based learning programs) and personalised approaches (e.g. social networks, opinion leaders, personal introductions and education in practice) [9]. So far, dissemination strategies have shown a heterogeneous low to moderate effect in implementing interventions [10–13]. For optimal dissemination, it is essential to adapt dissemination strategies to characteristics of the targeted individuals and the context in which they work [14]. *Information-seeking behaviour* refers to purposive seeking for information to satisfy some goal [15]. In physicians, educational meetings, websites and exchanges with colleagues are common sources [16, 17]. In a survey study with GPs in Denmark [18] medical websites, medication information websites and GP colleagues were the most frequently reported information sources. Medical websites, refundable continuing medical education (CME) meetings and guidelines of the Danish College of General Practitioners (DCGP) were perceived as being most important. Non-refundable CME meetings and pharmaceutical sales representatives were perceived as being important only by a minority [18]. Information-seeking can be motivated by opportunity. Additional reported barriers include time pressures, inadequate technical skills and, in some cases, paid access, especially for online sources [17].

### Aim and research questions

This study aimed to explore GPs information-seeking behaviours regarding recommendations on ambulatory cardiovascular care in Germany. Thereby we want to provide insights into the factors, processes and mechanisms of information-seeking behaviour, that could inform future designs of implementation strategies. Information-seeking behaviour of GPs on evidence-based recommendations in cardiovascular care was chosen as a focus: Cardiovascular diseases (CVD) are the leading cause of death worldwide, causing a total of approximately 40% of all deaths in Germany [19]. Accordingly, cardiology is the specialist medical discipline with which general practitioners in Germany are most involved and share a high number of patients. In addition, cardiology has the advantage that recommended practices are reasonably evidence-based and recommendations for medication therapy for heart failure were up-dated in 2016 and 2021 providing an example for new evidence within the field [20].

## Methods

### Study design

Reflecting the explorative nature of the research aim, a qualitative research design was chosen that encompassed semi-structured interviews with GPs. This qualitative study was part of the three-year ExKoCare project, which examined cooperation networks in German primary cardiovascular care [21]. Methods were reported according to the Consolidated Criteria for Reporting qualitative Research (COREQ) [22]. Included quotes have been translated into English (CA, CU) with due diligence and are cited with indication of participant number and transcript position.

### Study population

To be eligible for participation in the qualitative study, GPs needed to be participants in the ExKoCare Study, for which practising GPs in South Germany were recruited. GP practices from Baden-Wuerttemberg, Rhineland-Palatinate and Saarland were able to participate in the ExKoCare project and were invited via a fax letter. There were no specific inclusion criteria regarding the GP practices other than the declaration of consent by the practice owner [21].

### Recruitment and sampling

Invitation to participate in an interview were sent to all GPs included in the ExKoCare study. There was no additional relation between participants and researchers prior to the study. The study aimed at a structured purposive sampling strategy with regard to practice size, professional experience, age and gender to recruit a balanced sample of participants. Recruitment was carried out by the study team mainly via practice assistant (CA, PH, PT). Written information about the project, including researchers involved, and contact form to be returned via fax or e-mail were mailed to potential recruits. All interested GPs were contacted by phone. After receiving signed consent, date and time for the telephone interviews were confirmed individually. Reimbursement was offered to all participants to compensate for their time.

### Data collection

Semi-structured guide-based telephone interviews were conducted by three members of the study team (CA, PH, PT). All interviewers (two females, one male) had a background in health services research, were experienced interviewers and conducted interviews either from their workplace or from their home office. Participants were interviewed at home or at their workplace. To our knowledge no other people besides participants and researchers were present during the interviews. Based on the pre-defined research questions, the research team (CA, PH, PT, CU, MW) developed a study-specific interview guide in an iterative process of collecting, discussing and subsuming appropriate questions and wording. The interview-guide included twelve open-ended questions on four topics: (a) pathways of cardiovascular care, (b) coordination of care inside and outside of general practices, (c) patterns of orientation and (d) innovations and guidelines (see additional file 1). Interview topics were based on the research questions, existing research and preliminary results of the quantitative study within the ExKoCare project. While topics of the interview were set, the interview guide comprised open-ended questions to elicit subjective information form participants. A pilot interview with a GP from our research department was conducted. Adjustments were made throughout data collection where considered appropriate. All interviews were digitally recorded and transcribed verbatim. No field notes were made. Transcripts were not returned to participants for comments due to time restrictions of participants. All data collected in interviews were pseudonymized, digitally saved and stored on secure servers at the Department of General Practice and Health Services Research, University Hospital, Heidelberg, Germany.

### Data analysis

For a selection of interviews short memos were written and discussed within the broader research team (CA, PH, PT, CU) for preliminary familiarisation. Interview data was then analysed using thematic analysis [23] by two researchers (CA, CU), both female and with prior experience in qualitative methods: In a first step, open coding was used for data familiarisation. Successively, codes were developed and modified. In a second step, focused thematic coding was applied checking established themes against new data. Memos on interviews and theme development were written throughout data analysis. Within qualitative analysis, themes often emerge across topics of the interview guide. All interviews were coded by two researchers (CA, CU), who regularly discussed data and derived themes to ensure intercoder congruity and to achieve consensus. Analysis was reflected within the research team (CA, PH, PT, CU, MW). All data were analysed until consistency of findings enabled assessment of data sufficiency and thematic saturation [24]. The software package MAXQDA, Analytics Pro 2018 (Release 18.2.0, VERBI GmbH, Berlin, Germany) was used for the transcription of interviews, data management, and to facilitate sorting.

## Results

### Participants characteristics and overview

In total, 27 semi-structured guide-based telephone interviews with GPs were conducted between June and November 2021. Ten of the participating GPs were female and 17 males, working in 21 single-handed practices and 6 group practices (for detailed sample description see Table 1). Interviews were between 11:20 and 36:39 min with a mean duration of 21:26 min. All approached GPs participating in the ExKoCare study agreed to be interviewed and gave a written informed consent.

**Table 1:** Sample description

Characteristics	Numbers (N = 27)
Gender	10 female, 17 males
Age	Mean: 58.6 years (min: 37 years, max: 73 years)
Specialist specialty	Primary care internists: 5 General medicine: 21 Unknown: 1
Practice holder since	Mean: 19.3 years (min: 1 year, max: 39 years)
Practice type	21 single-handed practices, 6 group practices
Practice size: number of physicians per practices	One physician: 18 Two physicians: 8 Three physicians: 1
Practice size: total staff per practice	Two persons: 2 Three persons: 4 Four persons: 4 Five persons: 7 Six persons: 5 Seven persons: 3 15 persons: 1
Location of practice	Villages (under 5,000 inhabitants): 9 Small town (5,000–19,999 inhabitants): 3 Middle town (20,000–100,000): 14 City (bigger 100,000): 1

*Note to specialist specialty: In Germany, internists in ambulatory care usually work as GPs. However, their medical training has a different focus than that of GPs with a background in primary care*

The analysis of the interview data provided in-depth understanding of GPs information-seeking behaviour on (new) cardiological evidence. When asked, how they learn about new development or recommendations in the field of cardiology, GPs reported both generic information behaviour and casuistic information-seeking (see Table 2), the first referring to strategies GPs generally apply to keep up with medical developments, such as new medication, and the second referring to purposeful information exchange regarding individual patients, such as referral letters. The second strategy was also used to keep up with medical developments in general.

**Table 2:** Types of information behaviour relating to cardiological evidence-based recommendations in general practices

	Generic information behaviour	Casuistic information-seeking
Occasion of information-seeking behaviour	Regular events or behaviour	− Event driven − motivated by a specific patient case
Significance of cardiological evidence as a topic	(Coincidental) topic among others	Primary topic
Direction of information diffusion	Information dissemination from senders (e.g. journals, guideline, cardiologist)	Information-seeking of GPs
Role of GPs	GPs as a group, recipients	Initiated by individual GPs
Objective of information-seeking behaviour	Generic information on cardiovascular innovations	Specific patient-related information on cardiovascular innovations
Addressing of patients	Patients as a collective	Patient as individual
Sources	− Continuing medical education − Professional media (e.g. journals) − Pharmaceutical representatives − Participation in research studies	− Consultations of guidelines − Information from physicians’ letters (discharge letters)

GPs: general practitioners

### Generic information behaviour

Across the interviews, acquisition of additional generic cardiological evidence was describe as embedded in general information behaviour: Not specially targeted towards cardiology, this included (a) continuing education events, (b) reading professional periodicals and newsletters, (c) visits from pharmaceutical representatives and seldomly (d) participation in research studies.

#### A) Continuing medical education

Continuing medical education trainings were mentioned as activities where GPs encounter new cardiological evidence in most interviews. These included singular events organized by local physicians’ bodies and medical associations as well as events embedded in regular meetings, most prominently quality circles and (managed) care programs.

As a specific form of network meetings, quality circles were mentioned within some interviews. Quality circles are peer-led groups of 5–20 physician that meet regularly, usually quarterly. They are approved by the National Association of Statutory Health Insurance (NASHIP) to enhance professional exchange and reflection. While formalized to some extent, participants and content of meetings varied influencing contact with cardiological expertise: Within the interviews, GPs described GP-only quality circles in which cardiovascular diseases were sometimes topics of external expert talks or discussed within specific patient cases. One GP described in more detail how innovations are debated within this context:

*“Yes, quite a few things are discussed openly: ‘How do you do it in such a case?’ Or, in difficult cases, yes, when you can’t treat according to the guidelines; they ask: ‘How do you do it?’”. (I13: 95)*

Within cross-disciplinary quality circles, two had cardiologists as members and cardiovascular diseases were mentioned as a central topic of one meeting or part of an annual update by a cardiologist. Two of the circles were headed by cardiologists. In one case, regular dissemination of expert evidence was described as a continuous matter:

*“We have this GP quality circle, which is led by our cardiologist, who works very guideline-oriented and who always wants to keep us on track. So that we learn [how to do] it there.” (I27:89)*.

For some GPs, participation in structured care programmes facilitates information on new developments in cardiovascular care. Within these programmes, attendance in continuing medical education events are usually required about once a year. The description within the interviews showed that GPs counted on getting the information they need through these programmes. One interviewee described that, within disease management programme (DMP) meetings, specialist evidence is summarized for the primary care target audience:

*“Normally there is a DMP training once a year […] and there I will certainly learn the latest about the ESC [European Society of Cardiology] guideline […]. So, the classic DMP training […] actually summarized this subject […] for the GP colleagues. Everything is broken down [for us].“ (I23: 55–57)*.

In a similar manner participation in the family doctor program is described in another interview as a mandatory occasion to keep up with new developments in cardiovascular care:

*“And cardiovascular disease is always a topic there, because it is obligatory if you participate in the family doctor program [HZV]. Therefore, you are usually up to date. The cardiologists in private practice take part now and then and might give a lecture about the latest congress or developments.” (I19:25)*.

All in all, the significance attributed to continuing medical education ranged from little relevance to high relevance. One GP described selecting and conducting such events thoroughly as they provide a crucial professional basis:

*“I try to do my continuing medical education training really well. […] I am then able to look back on a good training that I did and can then work in a well-founded way so that I do not violate the guidelines.” (I15: 88)*.

However, within most interviews, continuing medical education events were seen as one way among others to keep up with new developments.

#### B) Professional media

Some GPs described reading professional periodicals, such as journals or newsletters, as a habit to remain informed about new developments. Explicitly mentioned were the “Deutsches Ärzteblatt” published by the German Medical Education Association and the NASHIP, the independent “arznei-telegramm” and the New England Journal of Medicine. One GP described how he tried to maintain reading journal articles that are eligible as a further education activity:

*“I don’t have the guidelines in my head at all, but that’s how it is. I try to read the German Medical Journal on the weekends when I have a little time. And there are always these CME articles, these continuing education articles, where you get three points. Until recently, I haven’t missed any of them […]. But now I can no longer keep up, because so many have been included. There are always very current clinical pictures discussed, from all specialties. At first you think: Oh, I’m not interested in that. But then, of course, you also come across cardiology guidelines and take note of them.” (I15:86)*.

Another GP described following medical newsletters from different sources, including commercial companies issuing regular information on a broad set of medical issues:

*“I also regularly receive medical newsletters from, um, Medscape, Doc-Check and others […]. And something like that [new guidelines] is always communicated as news. But, of course, you have to read it regularly. What I > laughs < admittedly not always manage to do.” (I19: 57)*.

Others, in contrast, underlined the pharma-critical nature of the journals and newsletters they read.

#### C) Pharmaceutical representatives

Within some interviews, the role of pharmaceutical sale representatives in providing information on new developments within the field of cardiology, especially on new drugs, were mentioned. While some stated a low significance as these representatives rarely visit their practice, in other interviews their role is described as more relevant: Pharmaceutical sales representatives are perceived to inform about innovations in a timely and sometimes insistent manner as one GP described:

*“I learn much from these pharmaceutical representatives who constantly come in here. They bring along [journal articles], that they sometimes co-authored, […]. For example, now for heart failure and therapy with SGLT2 [sodium-glucose linked transporter 2 inhibitors], so with, antidiabetics. […] So that, that is pressed in here. Yes, that is a lucrative business.” (I12: 61–63)*.

Besides academic journal articles, pharmaceutical sales representatives also provide direct information on guidelines and are involved in continuing medical education events as another GP reported:

*“Those who want to sell us something, beat a path at our door. So, we wouldn’t come up with a beta-blocker, would we? Well, it’s a high-quality drug, it will be sold by the pharmaceutical industry and then […], they are after you and bring along guidelines, make some effort. And they also offer further training and so on. […] These are, let’s say, scientifically oriented. So, the cardiologists from the big hospitals come here. Also, from the universities. So, this is not an advertising event, but rather an event that is also CME qualified […] yes, and that is then already correct in terms of content and meaningful.” (I24: 53–57)*.

Within the interviews, pharmaceutical representatives were described to offer a convenient and timely way to be updated on new developments in the field. Pharma industry involvement in studies, journal articles and continuing medical education events was stated in the interviews, but said to be balanced by scientific quality and involvement of clinical experts. Within one interview a GP stressed in addition:

*“The recommendations of the general practitioner quality circles and the recommendations of the specialist are clearly more important than those of the pharmaceutical representatives. Because I know the intention.” (I27: 101)*.

#### D) Participation in research studies

Participation in research studies can also lead to look into new developments of the field, as mentioned by two GPs. One example are clinical studies conducted by hospitals, that ask for a continuation of the study treatment within primary care. The second example referred to the ExKoCare-study itself:

*“Patient recruitment for the study actually made me a bit more aware of the diagnosis of heart failure. […] That, especially in heart failure, one has to pay more attention specifically to the guideline-based therapy, because it is not so obviously often, isn’t it?” (I16: 93)*.

Participation in research studies being the exception, most GPs used several of these ways to acquire evidence of new developments in cardiology. Not specifically target at cardiological content, these strategies were embedded in their general information behaviour.

### Casuistic information-seeking

While generic information behaviour comprises encountering general evidence on innovation, specific information-seeking centres around individual patient’s cases. By cooperating with cardiologists, GPs routinely come across expert evidence. However, the interviewed GPs described treatment of patients with cardiovascular diseases as largely guided by routines with little relevance of innovation: Cardiologists mainly provided routine check-ups, confirming and/or advanced diagnosis. Still, in the context of specific patient cases, two sources of information-seeking were accentuated by GPs: (a) consulting guidelines and (b) reading physicians letters, such as hospitals’ discharge letters or cardiologist reports.

#### A) Consultation of guidelines

When treating patients with cardiovascular conditions, most interviewed GPs described cardiological guidelines along work experience as central reference points. Some GPs reported to actively seek information via guidelines not only as routine practice when new guidelines are issued but also when treating specific patients with (complex) cardiovascular needs. One GP, for example, described routinely checking current guidelines when deciding how to assess cardiologist recommendation:

*“They [the cardiologists] make a recommendation […]. [That is] their point of view. And I look at what they have recommended, whether this fits the case, whether it suits me better and then I discuss this […] with the patient. […]. Whether the therapy fits or must be optimized or reduced, that is all very individual, but still guideline-appropriate. That means: I always look at what the guideline recommendation is and also look at what the health insurance companies usually recommend, which services are covered, and also to avoid regress, that is also very important.” (I07: 32)*.

Within other interviews, GPs reported to consult current guidelines in special cases, for example in case of doubt, when questions remained open or previous explanations seemed insufficient. One GP named a specific example:

*“Yes, [guidelines] serve as an important clue or reference, that I can always reread. Especially when it comes to the question, ICD implantation, yes/no. […] I put it this way: I have the impression that the clinics often or sometimes ignore the guidelines and that I then rather say: ‘So now we take it down a notch and first do the therapy and see how recovery goes […].”(I04:35)*.

However, some GPs explicitly distanced themselves from actively consulting guidelines in specific patients’ cases. One GP pointed to the diverse nature of the patient collective in primary care:

*“The guidelines are integrated continuing medical education training courses. The continuing medical education articles […] are based on guidelines […]. But it’s not like I look them up when the patient comes in with heart failure. Well, I know what is [written] in the guidelines, ACE inhibitors, beta-blockers and so on. […]. However, I’m not always in line with the guideline. Definitely not. This is also due to the fact that I don’t know them by heart. I don’t have them at my desk and consult them. […] The next patient is not a heart failure patient; the next patient is a paediatric care where a child is two years old or an infant is three months old. [.] That does not work, that is not possible.” (I15: 94–98)*.

In any case, the adaption to the setting of general practice was seen as a necessity.

#### B) Information from physicians’ letters

Across the interviews, physicians’ letters were seen as the primary mode of communication between GPs and cardiologists. Within some interviews, reading physicians letter was seen as a way to learn about innovation: For one GP, changes in recommended treatments motivated to further inquiries on new developments:

*"You either read it or you get it [within a letter], if you haven’t missed it, when it says again: ‘If I show atrial fibrillation, atrial size according to this and that score, he will now get this and that.’ Then I think: 'Oh, something has changed again, I have to read carefully again’ […] So, eh, indirectly yes. So, I don’t call and say: ‘Listen, when will the new guidelines come out and what does it say about this and that?’ (I14: 109–115)*."

Another GP reported that new developments and guidelines were explicitly part of the physicians’ letters:

*“And this heart clinic [name] actually also writes in its findings when there is a new guideline or something like that: ‘According to ESC guideline’.” (I11:77)*.

Within another interview, personal dialogue about guidelines were seen as unnecessary, precisely because the role of the physicians’ letters:

*I: "Do you ever discuss guidelines in the team? Do you?"*

*GP: “No, not really. Not really […] I can see within the cardiologist’s letter what he recommends, whether he follows the guidelines.” (I03: 78–81)*.

While physicians’ letters were described mostly as providing helpful, reliable and justified information, a few GPs reported insufficient and delayed letters.

## Discussion

Across interviews, a wide individual variety in relevance and composition of information-seeking behaviour concerning evidence-based recommendations in primary cardiology care was observed. Two broad strategies of information-seeking behaviour in GPs could be distinguished: (a) generic information-seeking behaviour and (b) casuistic information-seeking. Concerning generic developments in the field, GPs were informed by both written and online sources such as journals and newsletters and interpersonal sources within context-adapted formats such as educational trainings and ‘personalized’ approaches by pharmaceutical representatives or for study participation. This confirms previous findings [14–16], with one exception: Exchange of information among GP-colleagues was rarely reported. This can be attributed to the fact, that most interviewed GP worked in single-handed practices as it is common in Germany [16]. Concerning casuistic information-seeking, individual patient cases served as an incentive to actively consult evidence-based recommendations such as guidelines.

In addition, some GPs reported that they also learned directly from the exchange on individual patients (using physicians’ letters) on medical developments in general. These findings are in line with a known preference for pragmatic learning styles that are based on practical experience and casuistry rather than abstract information such as guidelines [4]. Particularly within the statements on continuing medical education events and visits from pharmaceutical representatives, a latent awaiting attitude among GP became apparent: Interviewees were confident that information on important innovations will find their way to them in a timely manner. At the same time, this strategy relied on somewhat incidental sources as, e.g., content of quality circles depends on composition of its members, topics of research articles depend on the journals usually read and the nature of information depends on the individual stance on the pharmaceutical industry. Our findings are consistent with the concept of “mindlines” [3] when diagnosing a health problem, but also showed GPs’ adherence to established ways of information behaviour – especially regarding the consultation of printed and online sources. On the one hand this strategy provides efficiency in information-seeking, on the other hand it implies risks if it is used to remain informed about general medical developments (e.g. the information may be wrong or selective). Previous research showed, that guidelines of professional general practitioners associations – e.g. in Denmark [18] – were among most frequently used information sources. The lack of references to primary care bodies and guidelines in our data confirm effects of a fragmented knowledge infrastructure in German primary care [6].

Our study focused on information-seeking behaviour. The actual use of the information in clinical decision-making was not documented. The interview sample largely reflected the characteristics of GPs in Germany regarding practice size and type, professional experience, age and gender [2]. Within our data set, there was little indication that these characteristics mattered for the information behaviours. The sample size limits confirmation of previous findings in this regard [2, 18]. Nonetheless, the density of the qualitative data facilitated consistency of findings and sufficient illustration of identified topics, indicating thematic saturation and sufficient sample size [24]. The topic of cardiovascular care was chosen for the number of shared patients of GPs and cardiologists and the availability of evidence-based recommendations. Information-seeking behaviour was described by GPs as largely habitual and guided by routines, suggesting similar behaviour when topics from other disciplines are concerned. However, rarer or less familiar diseases might be associated with additional or different information-seeking strategies. Across interviews, our data showed that individual information-seeking behaviour was largely influenced by circumstances and context, especially time constrains and the diversity of the patients’ cases in primary care. There were some indications, that personal motivation, professional self-perception and attitudes towards clinical evidence were also influencing factors. To some degree, our findings support the relevance of local opinion leaders as role models and facilitators of change [4, 25], influencing selection and prioritization of knowledge [1]. In our sample, local opinion leaders were often cardiologists preparing cardiological knowledge for a primary care audience, thus making it applicable by adaptation and prioritization. This is in line with findings from the survey study of the ExKoCare project, where 75% of GPs named an opinion leader, most of whom were cardiologists [25]. While opinion leaders are often the starting point for the dissemination of information, they are instable sources [26] and tend to be monomorphic (separated leaders for different topics and might be hard to identify) [27], limiting the effectiveness and practicability of using them for dissemination of evidence-based recommendations.

On a conceptual level, our study started out as an investigation of information-seeking behaviour, a well-established and widely used concept in implementation sciences. Although external factors are included as influencing information-seeking behaviour, “information-seeking behaviour” as a psychological concept focuses on individual and internal cognitive processes. Our results, however, also stress the social nature of the information process in the uptake of evidence-based recommendation. Information practice, a concept drawing from sociological theory of practice (especially Pierre Bourdieu, Anthony Giddens and Theo Schatzki) and recently suggested within information science [28], proposes a framework through which the ways in which individuals engage with information can be explored as social and culture processes. Within this concept, addressing particular characteristics of a social site of information engagement is inherent – including (but not limited to) individual and organizational circumstances, professional norms and perceptions, media and source and (last not least) the available information landscape.

## Conclusion

Our study was carried out in Germany focussing on cardiovascular care, which has specific features: Exchange of information among and between colleagues may be lower than in other countries due to a fragmented knowledge infrastructure and a tradition of single-handed practices. Compared to other countries, both GPs with a background in primary care and internal medicine are relatively well-trained in internal medicine. Therefore, their relationship to cardiologist may differ from countries, where GPs are more broadly trained across different or other medical specialities. Involvement of pharmaceutical representatives may be larger than in other presumed less profitable areas.

All in all, a comparison with the literature nevertheless indicates a relevance of the results beyond the German context: Our research confirms – by and large – findings on information-seeking behaviour towards generic information. However, the findings also showed the importance of casuistic information-seeking: Specific patient cases can not only serve as examples to accommodate pragmatic learning style preference, but also as occasions for information-seeking. The results indicate, that future implementation strategies should focus on both dissemination of generic (new) evidence and facilitation of specific patient-related information-seeking. Both structure care programs and physicians’ letters seem promising areas: Care programs offer a chance to regularly inform GPs about new generic developments, while physicians’ letters can deliver those in relation to the respective individual case. Both are embedded in daily practice and routines, close to individual patients’ needs and ask little extra time for making them more likely to be taken up – thus addressing typical obstacles in dissemination. Our findings also indicate that, within a fragmented information landscape with little systematic reference to primary care standards and guidelines, information-seeking is easily influenced by coincidence and, occasionally, bias. Further competency development in primary care in such countries could facilitate consistency, regularity and relevance of evidence-based recommendation of various medical domains for practicing GPs. On a conceptual level, our study indicates that it might be fruitful to adopt the concept of information practice to systematically address the uptake of evidence-based recommendation not only as an individual, but also as a social and cultural process. Nevertheless, information seeking or information practice only addresses one aspect of the uptake of recommendations. For comprehensive and sustained implementation of recommended practices, vocational learning has to be understood as something that is not limited to acquire knowledge in medical school, but rather as an ongoing process of life-long learning that may be guided by professional bodies.

## Electronic supplementary material

Below is the link to the electronic supplementary material.

**Additional file 1:** Semi-structured interview guide

## Data Availability

All data generated and analysed in this study are stored on a secure server at the University Hospital Heidelberg, Germany, Department of General Practice and Health Services Research. Due to data protection and privacy regulations, data is not available publicly. De-identified sets of the data collected and analysed during this study can be made available by the corresponding author upon reasonable request.

## Abbreviations

CME: Continuing medical education
COREQ: Consolidated Criteria for Reporting qualitative Research
DCGP: Danish College of General Practitioners
DMP: Disease management programme
ESC: European Society of Cardiology
ExKoCare: Exploration of mechanisms that influence coordination and uptake of recommended cardiovascular care
GP: General practitioner
I: Interview
NASHIP: National Association of Statuary Health Insurance
