# Supplementary material for: Keeping up with evidence-based recommendations – a qualitative interview study with general practitioners in Germany on information-seeking behaviour in cardiovascular care
Source: BMC Prim Care. 2023 May 25;24:118. doi: 10.1186/s12875-023-02069-7 (PMC10214602; doi:10.1186/s12875-023-02069-7)
Supplement: Supplementary file 1 — Additional file 1: Semi-structured interview guide [file 12875_2023_2069_MOESM1_ESM.docx]

**Additional file 1**

**Semi-structured interview guide**

**Introduction**

- Presentation of the interviewer and the study aim
- Information on data storage and data protection
- Information that the participants can stop their participation at any time during the interview

1. **Pathways of cardiovascular care**
2. How do you proceed when you meet a cardiovascular patient for the first time?
3. How would you describe the group of patients with cardiovascular diseases that you see within your practice?
4. **Coordination of cardiovascular care inside and outside of general practice**
5. Within your team, who is involved in caring for patients with cardiovascular diseases

(e.g. integration of physician assistants, tasks of different groups)? How do you experience collaboration within your team regarding this patient group?

1. What are typical occasions/reasons for referring patients to cardiologists?
2. **Patterns of Orientation**
3. How do you experience coordination/cooperation with cardiologists regarding this patient group? What are the typical tasks of GPs and cardiologists, respectively?
4. To what extent do you follow cardiologists’ advice? Who is ’in charge’ of the treatment?
5. What kind of information do you exchange with cardiologists? How do you do this?
6. What do you think, what would ’optimal cooperation’ of GPs and cardiologists look like?
7. **Perspectives on innovations and guidelines**
8. How do you decide how to treat patients with cardiovascular diseases? What are these decisions based on (e.g. experience, guidelines, colleagues, education)?
9. What do you think, are medical guidelines/recommendations (on cardiovascular care) helpful? Can you give an example? When are guidelines problematic?
10. How do you hear about updates on guidelines/recommendations?
11. When you assess guidelines, do you consider other views (e.g. from colleagues, professional associations, health insurance)?

**Ending**

- Do you have further thoughts you would like to share? Is there anything you would like to add?
- Thank you for participating in this interview.
